# Increased frequency of light physical activity during midlife and old age buffers against cognitive declines

**DOI:** 10.1007/s10865-024-00478-2

**Published:** 2024-03-01

**Authors:** Jeremy M. Hamm, Kelly Parker, Margie E. Lachman, Jacqueline A. Mogle, Katherine A. Duggan, Ryan McGrath

**Affiliations:** 1 Department of Psychology, North Dakota State University, Fargo, ND USA; 2 Department of Psychology, Brandeis University, Waltham, MA USA; 3 Department of Psychology, Clemson University, Clemson, SC USA; 4 Department of Kinesiology, North Dakota State University, Fargo, ND USA

**Keywords:** Light physical activity, Healthy cognitive aging, Episodic memory, Executive functioning, Longitudinal change

## Abstract

Although it is well established that moderate-to-vigorous physical activity (MVPA) buffers against declines in cognitive health, less is known about the benefits of light physical activity (LPA). Research on the role of LPA is crucial to advancing behavioral interventions to improve late life health outcomes, including cognitive functioning, because this form of physical activity remains more feasible and amenable to change in old age. Our study examined the extent to which increases in LPA frequency protected against longitudinal declines in cognitive functioning and whether such a relationship becomes pronounced in old age when opportunities for MVPA are typically reduced. We analyzed 9-year data from the national Midlife in the United States Study (*n* = 2,229; *M*_age_ = 56 years, range = 33–83; 56% female) using autoregressive models that assessed whether change in LPA frequency predicted corresponding changes in episodic memory and executive functioning in middle and later adulthood. Increases in LPA frequency predicted less decline in episodic memory (β = 0.06, *p* = .004) and executive functioning (β = 0.14, *p* < .001) over the 9-year follow-up period, even when controlling for moderate and vigorous physical activity. Effect sizes for moderate and vigorous physical activity were less than half that observed for LPA. Moderation models showed that, for episodic memory, the benefits of increases in LPA frequency were more pronounced at older ages. Findings suggest that increases in LPA over extended periods of time may help slow age-related cognitive declines, particularly in later life when opportunities for MVPA are often diminished.

## Introduction

Consistent evidence shows that physical activity is a critical health behavior linked to reduced risk of age-related chronic disease, functional impairment, and cognitive decline or impairment (Blondell et al., 2014; Fanning et al., 2017; Hoffmann et al., 2021; Miller et al., 2000; Warburton et al., 2010). For example, Hamer et al. (2018) found that higher baseline levels of moderate-to-vigorous physical activity (MVPA) predicted slower rates of 10-year decline in central indicators of healthy cognitive functioning (episodic memory, executive functioning) in a national UK sample of over 10,000 middle-aged and older adults. Although the relationship between MVPA and cognitive aging is well-established, less is known about the potential cognitive benefits of light physical activity (LPA). There is an urgent need for research on health behaviors such as LPA with the potential to support healthy cognitive aging because rates of dementia are expected to triple by 2060, unless there are major scientific advances in modifiable factors that can be targeted using evidence-based interventions (Matthews et al., 2019).

Light physical activities have metabolic equivalent of task (MET) values of 1.6-3 and include light walking, light sweeping, folding laundry, and washing dishes (Ainsworth et al., 1993; Mansoubi et al., 2015). Questions remain about the extent to which such LPAs can protect against longitudinal declines in cognitive functioning and whether such a relationship may become pronounced in old age when opportunities for MVPA are often limited. Research in this area is crucial because LPA remains more feasible, ingrained in everyday activities, and modifiable in late life, which may make it an ideal behavioral mechanism amenable to intervention (Chipperfield, 2008; Chipperfield et al., 2008; Erlenbach et al., 2021; Trinh et al., 2022). The present study therefore examined (a) whether longitudinal changes in LPA predicted 9-year trajectories of cognitive functioning and (b) if this association differed across the adult lifespan.

Few longitudinal studies have examined the role of LPA in buffering against age-related cognitive declines. Those that have largely focused on the relationship between LPA and risk of cognitive impairment in late life based on coarse cutoff scores derived from the Mini-Mental State Examination (no cognitive impairment vs. cognitive impairment). Laurin et al. (2001) found that higher baseline levels of LPA predicted reduced risk of 5-year cognitive impairment in a national sample of older Canadians. Similar results were observed in subsequent studies that found older adults with higher LPA were at lower risk of cognitive impairment over follow-up periods of 2–8 years (Lee et al., 2013; Lytle et al., 2004; Stubbs et al., 2017; Yaffe et al., 2001). These studies provided initial support for the cognitive benefits of LPA but were limited by their reliance on relatively coarse, binary indicators of cognitive functioning that classified individuals as either having cognitive impairment or not.

Several longitudinal investigations built on this prior work by exploring whether LPA predicted subclinical declines in more sensitive, continuous indicators of healthy cognitive aging that included episodic memory and executive functioning. Thibeau et al. (2016, 2017) provided indirect evidence for this relationship in showing that everyday physical activity (a combination of light, moderate, and vigorous activity) was associated with less decline in late life executive functioning. In contrast, Whitaker et al. (2021) found that substituting amount of sedentary time with LPA was not associated with reduced rates of decline in episodic memory or executive functioning in a sample of adults in early midlife (aged 38–50). Collectively these findings provide mixed evidence that LPA may potentially buffer against subclinical cognitive declines, but a systematic evaluation of this relationship across the adult lifespan is lacking.

Questions also remain regarding whether *maintaining* or *increasing* LPA over time may help to slow age-related declines in episodic memory and executive functioning. Observational work of this type is critical because it may identify LPA as a *modifiable* (i.e., intervenable) lifestyle factor related to cognitive decline. Research has yet to address this issue because past studies have focused on the role of LPA assessed at a single time point (e.g., Laurin et al., 2001; Lee et al., 2013; Lytle et al., 2004; Stubbs et al., 2017; Thibeau et al., 2016; Whitaker et al., 2021). This approach failed to capture ecological realities and critical long-term trends wherein LPA can decline, remain stable, or even increase as people age and encounter shifting developmental opportunities (e.g., increased time) and constraints (e.g., functional limitations; Hamm et al., 2023; Heckhausen et al., 2019). Research is needed to examine whether longitudinal *changes* in LPA predict corresponding shifts in cognitive functioning.

The benefits of LPA for cognitive aging may also differ across the adult lifespan, but little is known about whether this relationship depends on age. Past research provided some indirect evidence that the cognitive benefits of LPA may be stronger in late life. For instance, the longitudinal studies that found LPA was associated with reduced risk of cognitive decline were based on older samples aged at least 60 years (Laurin et al., 2001; Lee et al., 2013; Lytle et al., 2004; Stubbs et al., 2017; Thibeau et al., 2016, 2017; Yaffe et al., 2001). In contrast, Whitaker (2021) did not observe a relationship between LPA and cognitive functioning among a younger sample in early midlife (aged 38–50). The only study to explicitly examine the moderating role of age found that everyday physical activity was more strongly associated with preserved cognitive functioning in young-old, but not old-old, adults (Thibeau et al., 2017). However, this study measured physical activity using a combination of LPA, moderate physical activity (MPA), and vigorous physical activity (VPA) in a sample that included only older adults (aged 53–95). Research is needed to test the moderating role of age in an adult lifespan sample using a measure of LPA that is clearly distinguished from MPA and VPA.

The present study used 9-year data from the national Midlife in the United States Study to examine whether changes in LPA predicted corresponding trajectories of cognitive functioning beyond more vigorous forms of physical activity. We focused on longitudinal changes in central indicators of cognitive functioning shown to be sensitive to early age-related declines: episodic memory and executive functioning (Hughes et al., 2018). We expected increases in LPA frequency to predict less decline in episodic memory and executive functioning over the 9-year follow-up. We also evaluated whether the longitudinal relationship between LPA and cognitive functioning was pronounced in old age. Our premise was that maintaining or increasing LPA frequency may become paramount in buffering against cognitive declines as individuals age and encounter increasing constraints that can limit their capacity to engage in MVPA. Preliminary analyses were also conducted to supplement our main research questions by examining whether age-related disparities in physical activity were smallest for LPA.

## Method

### Participants and procedures

We examined our research questions using data from the Midlife in the United States National Longitudinal Study of Health and Well-being (MIDUS). A detailed summary of MIDUS can be found elsewhere (Brim et al., 2004; Ryff et al., 2017). Briefly, MIDUS is an ongoing national study of U.S. adults who were 25–75 years old at baseline assessment (1995–2013). Baseline data were assessed in 1995 (Wave 1; *n* = 7,108), and all willing participants were reassessed in 2004 (Wave 2; *n* = 4,963) and 2013 (Wave 3; *n* = 3,294). The current study focused on participants from the second and third waves because LPA and cognitive functioning were not assessed at the first wave. At both Waves 2 and 3, survey data on our predictor variables (LPA, MPA, VPA) were assessed approximately 1 month prior to data on our cognitive outcome measures (episodic memory, executive functioning).

Inclusion criteria for the present study were that participants provided data at Waves 2 and 3 on our focal predictor measures of LPA, MPA, or VPA and at least one of our primary outcome measures of episodic memory or executive functioning. These criteria allowed us to examine how 9-year changes in different intensities of physical activity were associated with corresponding trajectories of cognitive functioning. At Wave 2, the analyzed sample (*n* = 2,229) had a mean age of 56 ± 11 years (range = 33–83), was 56% female and 95% White, had an average household income of $76,173, and 71% had some postsecondary education. As is typical in longitudinal studies (Lindenberger et al., 2001; Radler & Ryff, 2010), participants in the analyzed sample (who provided longitudinal data at Waves 2 and 3) were more likely to be younger, female, have higher education and income, have fewer functional limitations, to be more physically active, and to have higher episodic memory and executive functioning (*p*s = 0.001-0.039). The magnitudes of these differences were small (*d*s = 0.06-0.37; Cohen, 1988). A detailed summary of attrition in MIDUS can be found elsewhere (Hughes et al., 2018; Radler & Ryff, 2010).

### Study measures

#### Physical activity

Frequency of light (LPA), moderate (MPA), and vigorous physical activity (VPA) were assessed with 18 items at Waves 2 and 3 using a 6-point scale (1 = *several times a week or more*, 6 = *never*). We reverse coded all items so that higher scores reflected more frequent physical activity. Participants reported how frequently they participated in LPA (e.g., easy walking, light housework, fishing), MPA (e.g., brisk walking, low impact aerobics), and VPA (e.g., running, lifting heavy objects) during summer and winter and in home, work, and leisure settings. Items thus captured frequency of light, moderate, and vigorous physical activity across multiple domains and seasons.

We created continuous measures of LPA, MPA, and VPA at Waves 2 and 3 following the approach developed by Lachman et al. (Cotter & Lachman, 2010; Robinson & Lachman, 2018). Participants’ highest LPA score from either the home, work, or leisure domain in summer was averaged with their highest LPA score from either the home, work, or leisure domain in winter. In this way, participants who engaged in regular light activity during leisure time but not at work or home (or vice versa) were still scored as being frequently engaged in LPA. The same approach was used to create MPA and VPA scores.^1^ As recommended by Cohen, Cohen, West, and Aiken ( 2013) when using two-wave longitudinal data, we subsequently generated our primary predictor measures of regressed (residualized) change in LPA, MPA, and VPA frequency by regressing Wave 3 scores on the corresponding baseline (Wave 2) levels of each measure. Residuals from these analyses reflected regressed change that statistically partialed out variance due to baseline levels in each measure of physical activity (Maxwell et al., 2017; Tennant et al., 2022). We saved these residuals and used them as indicators of regressed, longitudinal change in LPA, MPA, and VPA frequency (Cohen et al., 2013). See Table 1, Table 2, and Table S1 for a summary of the sample characteristics and zero-order correlations between the study variables.

**Table 1:** *Descriptive Characteristics of the Participants*

Variable	M ± SD or n (%)	Range
Age^b^	55.62 ± 11.13	33–83
Sex^a^		
Male	979 (43.9%)	
Female	1250 (56.1%)	
Race^a^		
White	2119 (95.1%)	
Black	53 (2.4%)	
Native American	5 (0.2%)	
Asian or Pacific Islander	9 (0.4%)	
Other	27 (1.2%)	
Multiracial	16 (0.7%)	
SES^b^	0.09 ± 0.69	-1.95-2.53
ADL limitations^b^	1.67 ± 0.81	1–4
LPA^b^	5.24 ± 1.31	1–6
MPA^b^	4.26 ± 1.70	1–6
VPA^b^	3.52 ± 1.86	1–6
Episodic memory^b^	0.11 ± 0.91	-2.10-3.64
Executive functioning^b^	0.13 ± 0.64	-2.08-2.34
LPA^c^	5.15 ± 1.40	1–6
MPA^c^	4.21 ± 1.77	1–6
VPA^c^	3.50 ± 1.92	1–6
Episodic memory^c^	-0.01 ± 0.99	-2.94-3.64
Executive functioning^c^	-0.13 ± 0.73	-5.63-2.02

Note. SES = socioeconomic status. ADL = activities of daily living (limitations). LPA = light physical activity. MPA = moderate physical activity. VPA = vigorous physical activity

^a^Wave 1 ^b^Wave 2 ^c^Wave 3

**Table 2:** *Zero-Order Correlations for the Main Study Variables*

Variable	1	2	3	4	5	6	7	8	9	10	11	12	13	14	15	
1. Age^b^	–															
2. Sex (female)^a^	0.00	–														
3. Race (minority)^a^	− 0.01	0.03	–													
4. SES^b^	− 0.10	− 0.18	− 0.01	–												
5. ADL limitations^b^	0.28	0.17	0.01	− 0.25	–											
6. LPA^b^	− 0.11	0.07	− 0.08	0.16	− 0.14	–										
7. MPA^b^	− 0.17	− 0.07	− 0.05	0.24	− 0.27	0.49	–									
8. VPA^b^	− 0.26	− 0.12	− 0.05	0.22	− 0.25	0.31	0.64	–								
9. Episodic memory^b^	− 0.27	0.24	− 0.04	0.15	− 0.12	0.16	0.14	0.13	–							
10. Executive functioning^b^	− 0.39	− 0.13	− 0.12	0.36	− 0.24	0.16	0.23	0.22	0.37	–						
11. ΔLPA^bc^	− 0.18	0.04	− 0.02	0.11	− 0.16	− 0.01	0.15	0.14	0.10	0.14	–					
12. ΔMPA^bc^	− 0.20	− 0.08	− 0.01	0.15	− 0.21	0.05	0.00	0.17	0.06	0.12	0.47	–				
13. ΔVPA^bc^	− 0.18	− 0.09	0.01	0.13	− 0.16	0.05	0.09	0.01	0.06	0.12	0.28	0.53	–			
14. ΔEpisodic memory^bc^	− 0.28	0.14	− 0.04	0.11	− 0.12	0.09	0.09	0.08	0.00	0.19	0.13	0.07	0.07	–		
15. ΔExecutive functioning^bc^	− 0.30	− 0.03	− 0.03	0.06	− 0.13	0.05	0.08	0.11	0.05	0.00	0.18	0.11	0.11	0.23	–	

Note. EM = episodic memory. SES = socioeconomic status. ADL = activities of daily living (limitations). LPA = light physical activity. MPA = moderate physical activity. VPA = vigorous physical activity. Δ = regressed change. *n* range = 1996–2229. All correlations ≥. |05| are significant at *p* < .05

^a^Wave 1 ^b^Wave 2 ^c^Wave 3

#### Cognitive function

The Brief Test of Adult Cognition by Telephone (BTACT) was used to assess episodic memory and executive functioning at Waves 2 and 3 (Lachman & Tun, 2008; Tun & Lachman, 2006). Previous research with middle-aged and older adults has shown the BTACT to be a reliable and valid measure of central dimensions of cognition involving episodic memory and executive functioning (Hamm et al., 2020; Lachman et al., 2014; Tun & Lachman, 2006). A detailed summary of the BTACT can be found elsewhere (Hughes et al., 2018; Lachman et al., 2010, 2014).

Briefly, the BTACT battery includes two cognitive tests to assess episodic memory and five tests to assess executive functioning (Lachman et al., 2014). Episodic memory was assessed using immediate and delayed recall tasks (free recall of 15 words). Executive functioning was assessed using measures of inductive reasoning (completing patterns in a number series), category verbal fluency (number of animal names produced in one minute), working memory span (backward digit span), processing speed (number of digits produced counting backwards from 100 in 30 s), and attention switching and inhibitory control (Stop and Go Switch Task). The Stop and Go Switch Task comprised a reaction time test involving normal (respond GO to stimulus GREEN and STOP to stimulus RED) and reverse conditions (respond STOP to stimulus GREEN and GO to stimulus RED) (Tun & Lachman, 2008). For the executive functioning measure, we used a recommended filter that retained data for only participants with valid scores on the Stop and Go Switch Task (Lachman et al., 2014; Tun & Lachman, 2008). Valid scores were those in which there were no technical malfunctions, the participant understood the task, and the participant was not distracted by external events.

Measures of episodic memory and executive functioning factors were calculated by averaging the standardized values of their respective subtests at each wave (Hughes et al., 2018). We generated our primary outcome measures of regressed (residualized) change in episodic memory and executive function by regressing Wave 3 scores on the corresponding baseline (Wave 2) levels of each measure (Cohen et al., 2013; Maxwell et al., 2017; Tennant et al., 2022). Residuals from these analyses were saved and used as indicators of regressed, longitudinal change in episodic memory and executive functioning (Cohen et al., 2013). We note that scores of 0 on our regressed change measures roughly reflect average sample rates of 9-year decline in episodic memory (raw decline *M* = − 0.125) and executive functioning (raw decline *M* = − 0.259).

#### Demographic covariates

Age, sex, race, socioeconomic status, and functional limitations are well-established correlates of physical activity and cognitive functioning and were included as covariates in the main analyses (Dixon & Lachman, 2019; Hughes et al., 2018; Lachman et al., 2014; Robinson & Lachman, 2018; Tran et al., 2014). Age in years (*M* = 55.62, *SD* = 11.13) was assessed at Wave 2. Sex (1 = *male*, 2 = *female*; 56% female) and race (0 = *white*, 1 = *non-white*; 95% white) were assessed at Wave (1) Three self-report measures of socioeconomic status (SES) were assessed at Wave 2: level of formal education completed (1 = *no school or grade school*, 12 = *doctoral degree*; *M* = 7.57, *SD* = 2.51), total household income in US dollars (*M* = 76,173, *SD* = 60,557), and perceived SES using the reverse-coded MacArthur Scale of Subjective Social Status (1 = *top rung*, 10 = *bottom rung*; *M* = 6.59, *SD* = 1.79). Because the three SES indicators were positively correlated (*r*s = 0.13-0.35), we computed a composite SES score by first *z*-standardizing and then averaging the *z*-scored measures (*M* = 0.09, *SD* = 0.69) (Hamm et al., 2021; Wrosch et al., 2018). Activities of daily living (ADL limitations) were assessed at Wave 2. Participants reported the extent to which health limited their ability to perform 7 ADLs using a 4-point scale: lifting or carrying groceries; climbing several flights of stairs; bending, kneeling, or stooping; walking more than a mile; walking several blocks; vigorous activities (e.g., running); and moderate activities (e.g., vacuuming) (1 = *a lot*, 4 = *not at all*). Scores were reverse coded so that higher scores reflected greater functional limitations (*M* = 1.69, *SD* = 0.81).

### Rationale for analyses

#### Preliminary analyses

Preliminary repeated measures ANOVAs assessed age differences in light (LPA), moderate (MPA), and vigorous physical activity (VPA) at Wave 2. These analyses enabled us to examine whether older adults were able to better maintain LPA relative to their younger peers. Age was treated as a between-subjects factor with three levels: young adults aged 33–39 (*n* = 161), middle-aged adults aged 40–59 (*n* = 1246), and older adults aged 60+ (*n* = 794). Physical activity was treated as a within-subjects factor with three levels: frequency of LPA, MPA, and VPA. We thus employed a 3 by 3 Age (young, middle-aged, old) x Physical Activity (LPA, MPA, VPA) repeated measures ANOVA to assess whether age-related losses in physical activity would be smallest for light physical activity.

#### Main analyses

The main analyses involved autoregressive, OLS regression models that were conducted in a stepwise fashion using SPSS v.28. Step 1 models assessed the extent to which regressed changes in frequency of LPA, MPA, and VPA predicted 9-year, regressed change in cognitive functioning (main effect models). Step 2 models incorporated interaction terms with age to assess whether the influence of changes in LPA, MPA, and VPA frequency on changes in cognitive functioning differed across the adult lifespan (interaction effect models). All models controlled for age, sex, race, socioeconomic status, functional limitations, and baseline levels of each predictor and outcome measure (i.e., autoregressive effects). The predictor and outcome variables in our models thus reflected regressed change in physical activity frequency and cognitive functioning rather than raw change or gain scores, which can produce misleading results (Cohen et al., 2013; Maxwell et al., 2017; Tennant et al., 2022). As recommended by Cohen et al. (2013) and Tennant et al. (2022) when using two-wave longitudinal data, this autoregressive approach enabled us to examine whether changes in LPA predicted corresponding shifts in cognitive functioning while partialing out variance due to baseline levels of our predictors and outcomes. All continuous variables were left in their original metric (only sex and race were treated as categorical variables) (Hayes, 2017). Standardized and unstandardized regression coefficients are presented for all models.

## Results

### Preliminary analyses: age differences in light, moderate, and vigorous physical activity

Results from a repeated measures ANOVA yielded main effects of age (*F*_2, 2198_ = 49.58, *p* < .001) and physical activity (*F*_2, 2197_ = 441.01, *p* < .001, Wilk’s Lambda = 0.714). The age main effect was consistent with previous research in showing that, overall, older adults engaged in physical activity less frequently than their peers in young adulthood and midlife (Bisson & Lachman, 2023; Takagi et al., 2015; Varma et al., 2017). The physical activity main effect indicated that participants engaged in LPA more frequently than MPA or VPA.

**Fig. 1:**
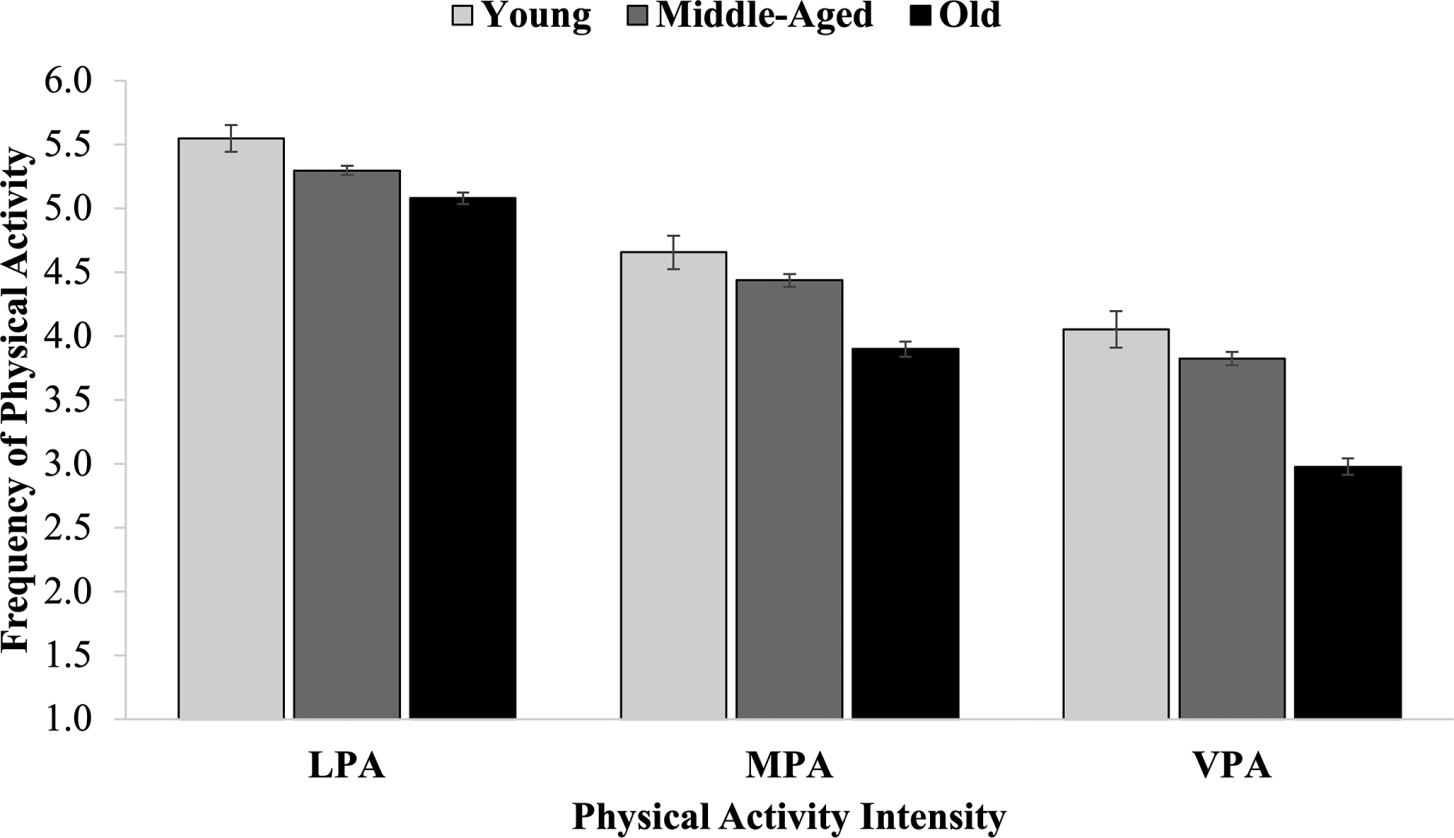
Age x Physical Activity interaction on frequency of physical activity. LPA = light physical activity. MPA = moderate physical activity. VPA = vigorous physical activity

The Age x Physical Activity interaction was also significant (*F*_4, 4394_ = 13.99, *p* < .001, Wilk’s Lambda = 0.975). Simple main effect contrasts revealed an expected pattern that showed age-related losses in physical activity were substantially smaller for LPA than for MPA or VPA (see Fig. 1). For MPA and VPA, large differences emerged between older adults and their younger (*M*_diffs_ = 0.75, 1.08) and middle-aged peers (*M*_diffs_ = 0.54, 0.85). This discrepancy was markedly reduced for LPA such that older adults were able to maintain more comparable levels of LPA relative to their peers in young adulthood (*M*_diff_ = 0.47) and midlife (*M*_diff_ = 0.22).

### Step 1: light, moderate, and vigorous physical activity as predictors of 9-year change in cognitive functioning (main effect models)

Step 1 OLS regression models assessed the extent to which regressed changes in LPA, MPA, and VPA frequency predicted corresponding 9-year regressed changes in episodic memory and executive functioning. LPA, MPA, and VPA were first entered separately to examine the influence of each physical activity indicator irrespective of the others. Subsequent OLS regression models entered the predictors simultaneously to assess the extent to which changes in LPA, MPA, and VPA frequency uniquely predicted corresponding 9-year changes in episodic memory and executive functioning. All models controlled for age, sex, race, socioeconomic status, functional limitations, and baseline levels of the predictors and outcomes (i.e., autoregressive effects).

#### Episodic memory

Results of separate regression models showed that only increases in LPA frequency predicted less decline in episodic memory over the 9-year follow-up (β = 0.06, *b* = 0.04, *SE* = 0.013, *p* = .004; see Table 3, Model 1a). Increases in MPA and VPA frequency were not associated with slower declines in episodic memory (*p*s > 0.05; see Table 3, Models 1b and 1c). Results of subsequent regression models with LPA, MPA, and VPA entered simultaneously yielded similar results. Only increases in LPA frequency uniquely predicted less decline in episodic memory (β = 0.06, *b* = 0.04, *SE* = 0.015, *p* = .010; see Table 3, Model 1d). Sensitivity analyses that replaced MPA and VPA with a composite indicator of MVPA replicated the main results (see Table S2). Increases in MVPA frequency did not predict change in episodic memory in either the separate model or the simultaneous model (*p*s > 0.05). Only increases in LPA frequency uniquely predicted less decline in episodic memory in the simultaneous model (β = 0.06, *b* = 0.04, *SE* = 0.014, *p* = .004).

**Table 3:** *Main Effect Model Regression Coefficients for 9-Year Regressed Changes in Episodic Memory (Step 1)*

	Model 1a			Model 1b			Model 1c			Model 1d	
	β	*b* (*SE*)		β	*b* (*SE*)		β	*b* (*SE*)		β	*b* (*SE*)
Baseline EM	− 0.16^**^	− 0.15 (0.020)		− 0.15^**^	− 0.14 (0.020)		− 0.15^**^	− 0.14 (0.020)		− 0.16^**^	− 0.15 (0.020)
Age	− 0.28^**^	− 0.02 (0.002)		− 0.28^**^	− 0.02 (0.002)		− 0.29^**^	− 0.02 (0.002)		− 0.28^**^	− 0.02 (0.002)
Sex (female)	0.41^**^	0.34 (0.036)		0.42^**^	0.35 (0.036)		0.43^**^	0.36 (0.036)		0.41^**^	0.34 (0.036)
Race (minority)	− 0.19^*^	− 0.16 (0.077)		− 0.22^*^	− 0.18 (0.077)		− 0.23^*^	− 0.19 (0.076)		− 0.20^*^	− 0.17 (0.077)
SES	0.12^**^	0.14 (0.026)		0.12^**^	0.14 (0.026)		0.12^**^	0.14 (0.026)		0.11^**^	0.13 (0.026)
ADL limitations	− 0.05^*^	− 0.05 (0.023)		− 0.06^*^	− 0.06 (0.023)		− 0.06^*^	− 0.06 (0.023)		− 0.06^*^	− 0.06 (0.023)
Baseline LPA	0.04	0.02 (0.013)								0.04	0.02 (0.015)
ΔLPA	0.06^**^	0.04 (0.013)								0.06^*^	0.04 (0.015)
Baseline MPA				0.03	0.02 (0.010)					0.02	0.01 (0.015)
ΔMPA				0.01	0.00 (0.011)					− 0.02	− 0.01 (0.014)
Baseline VPA							0.01	0.00 (0.010)		− 0.02	− 0.01 (0.013)
ΔVPA							0.02	0.01 (0.010)		0.01	0.01 (0.012)

Note. EM = episodic memory. SES = socioeconomic status. ADL = activities of daily living (limitations). LPA = light physical activity. MPA = moderate physical activity. VPA = vigorous physical activity. Δ = regressed change in predictor. β coefficients are *z*-standardized except for the binary sex and race variables which have been left in the original metric to facilitate interpretation (Hayes, 2017). *b* coefficients are unstandardized. *n* range = 2153–2195

^*^p < .05; ^**^p < .01

#### Executive functioning

Results of separate regression models indicated that increases in LPA (β = 0.14, *b* = 0.05, *SE* = 0.008, *p* < .001), MPA (β = 0.05, *b* = 0.02, *SE* = 0.006, *p* = .016), and VPA frequency (β = 0.06, *b* = 0.02, *SE* = 0.006, *p* = .011) predicted less decline in executive functioning over the 9-year follow-up (see Table 4, Model 2a, 2b, 2c). However, the magnitude of MPA and VPA associations were less than half the size of those observed for LPA. Results of subsequent regression models with LPA, MPA, and VPA entered simultaneously showed that only increases in LPA frequency uniquely predicted less decline in executive functioning (β = 0.15, *b* = 0.05, *SE* = 0.009, *p* < .001; see Table 4, Model 2d). Sensitivity analyses that replaced MPA and VPA with a composite indicator of MVPA replicated the main results (see Table S3). Increases in MVPA frequency predicted less decline in executive functioning in the separate model (β = 0.07, *b* = 0.02, *SE* = 0.006, *p* = .003). However, only increases in LPA frequency uniquely predicted less decline in executive functioning in the simultaneous model (β = 0.14, *b* = 0.05, *SE* = 0.008, *p* < .001).

**Table 4:** *Main Effect Model Regression Coefficients for 9-Year Regressed Changes in Executive Functioning (Step 1)*

	Model 2a			Model 2b			Model 2c			Model 2d	
	β	*b* (*SE*)		β	*b* (*SE*)		β	*b* (*SE*)		β	*b* (*SE*)
Baseline EF	− 0.19^**^	− 0.14 (0.018)		− 0.19^**^	− 0.14 (0.018)		− 0.18^**^	− 0.13 (0.018)		− 0.19^**^	− 0.14 (0.018)
Age	− 0.33^**^	− 0.01 (0.001)		− 0.33^**^	− 0.01 (0.001)		− 0.33^**^	− 0.01 (0.001)		− 0.32^**^	− 0.01 (0.001)
Sex (female)	− 0.08	− 0.04 (0.021)		− 0.05	− 0.02 (0.021)		− 0.04	− 0.02 (0.021)		− 0.07	− 0.03 (0.021)
Race (minority)	− 0.22^*^	− 0.10 (0.046)		− 0.24^*^	− 0.11 (0.046)		− 0.22^*^	− 0.10 (0.46)		− 0.22^*^	− 0.11 (0.046)
SES	0.06^**^	0.04 (0.016)		0.06^**^	0.04 (0.016)		0.06^**^	0.04 (0.016)		0.06^*^	0.04 (0.016)
ADL limitations	− 0.03	− 0.02 (0.013)		− 0.03	− 0.02 (0.014)		− 0.04	− 0.02 (0.014)		− 0.03	− 0.02 (0.014)
Baseline LPA	0.03	0.01 (0.008)								0.03	0.01 (0.009)
ΔLPA	0.14^**^	0.05 (0.008)								0.15^**^	0.05 (0.009)
Baseline MPA				0.04	0.01 (0.006)					− 0.01	− 0.00 (0.009)
ΔMPA				0.05^*^	0.02 (0.006)					− 0.04	− 0.01 (0.008)
Baseline VPA							0.04	0.01 (0.006)		0.03	0.01 (0.007)
ΔVPA							0.06^*^	0.02 (0.006)		0.03	0.01 (0.007)

Note. EF = executive functioning. SES = socioeconomic status. ADL = activities of daily living (limitations). LPA = light physical activity. MPA = moderate physical activity. VPA = vigorous physical activity. Δ = regressed change in predictor. β coefficients are *z*-standardized except for the binary sex and race variables which have been left in the original metric to facilitate interpretation (Hayes, 2017). *b* coefficients are unstandardized. *n* range = 1975–2009

^*^p < .05; ^**^p < .01

#### Contextualized effect sizes

**Fig. 2:**
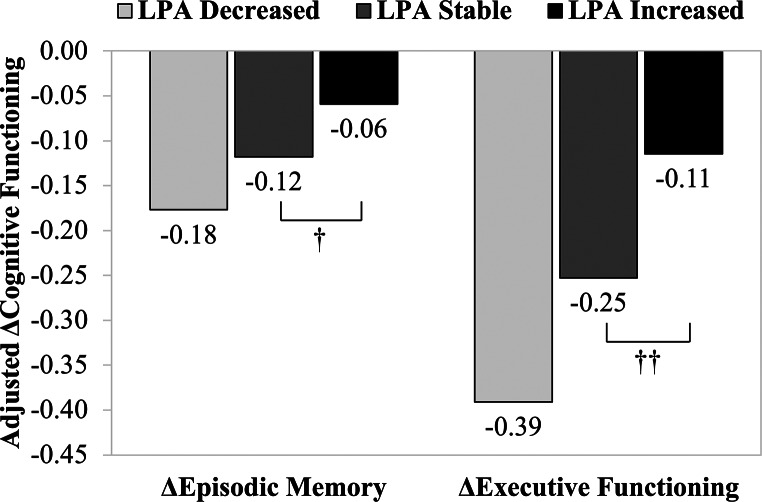
Contextualized effect sizes of ΔLPA on regressed changes in episodic memory and executive functioning. Estimates suggest rates of 9-year decline in episodic memory and executive functioning were respectively reduced by approximately 50% (-0.12 vs. − 0.06) and 55% (-0.25 vs. − 0.11) for those who increased their LPA by 1 *SD* relative to those whose activity levels remained stable. Predicted values were adjusted for average sample declines of − 0.13 units in episodic memory and − 0.26 units for executive functioning. LPA = light physical activity ^†^50% reduction in rate of decline; ^††^55% reduction in rate of decline

We generated predicted values (PVs) that adjusted for average sample declines of − 0.13 units in episodic memory and − 0.26 units in executive functioning (see Method) to contextualize the practical significance and effect size of changes in LPA frequency (see Fig. 2). Small but meaningful differences emerged in predicted episodic memory scores for participants whose LPA frequency declined by 1 *SD* (PV = − 0.177), remained stable (PV = − 0.117), or increased by 1 *SD* (-0.059). A similar pattern of differences was observed in predicted executive functioning scores for those whose LPA frequency declined by 1 *SD* (PV = − 0.391), remained stable (PV = − 0.253), or increased by 1 *SD* (-0.115). These estimates suggest that rates of 9-year decline in episodic memory were reduced by approximately 50% (-0.117 vs. − 0.059) and approached stability (minimal decline) for those who increased their LPA by 1 *SD* relative to those whose activity levels remained stable. Similarly, rates of decline in executive functioning were reduced by approximately 55% (-0.253 vs. − 0.115) for participants who increased their LPA by 1 *SD* relative to those who remained stable.

### Step 2: age-moderated effects of light, moderate and vigorous physical activity on 9-year change in cognitive functioning (interaction effect models)

Step 2 involved separate OLS regression models that incorporated interaction terms with age to assess whether the influence of changes in LPA, MPA, and VPA frequency on corresponding changes in cognitive functioning differed across the adult lifespan. Variables involved in the interaction terms were centered to facilitate interpretation. All models controlled for age, sex, race, socioeconomic status, functional limitations, and baseline levels of the predictors and outcomes (i.e., autoregressive effects).

#### Episodic memory

**Fig. 3:**
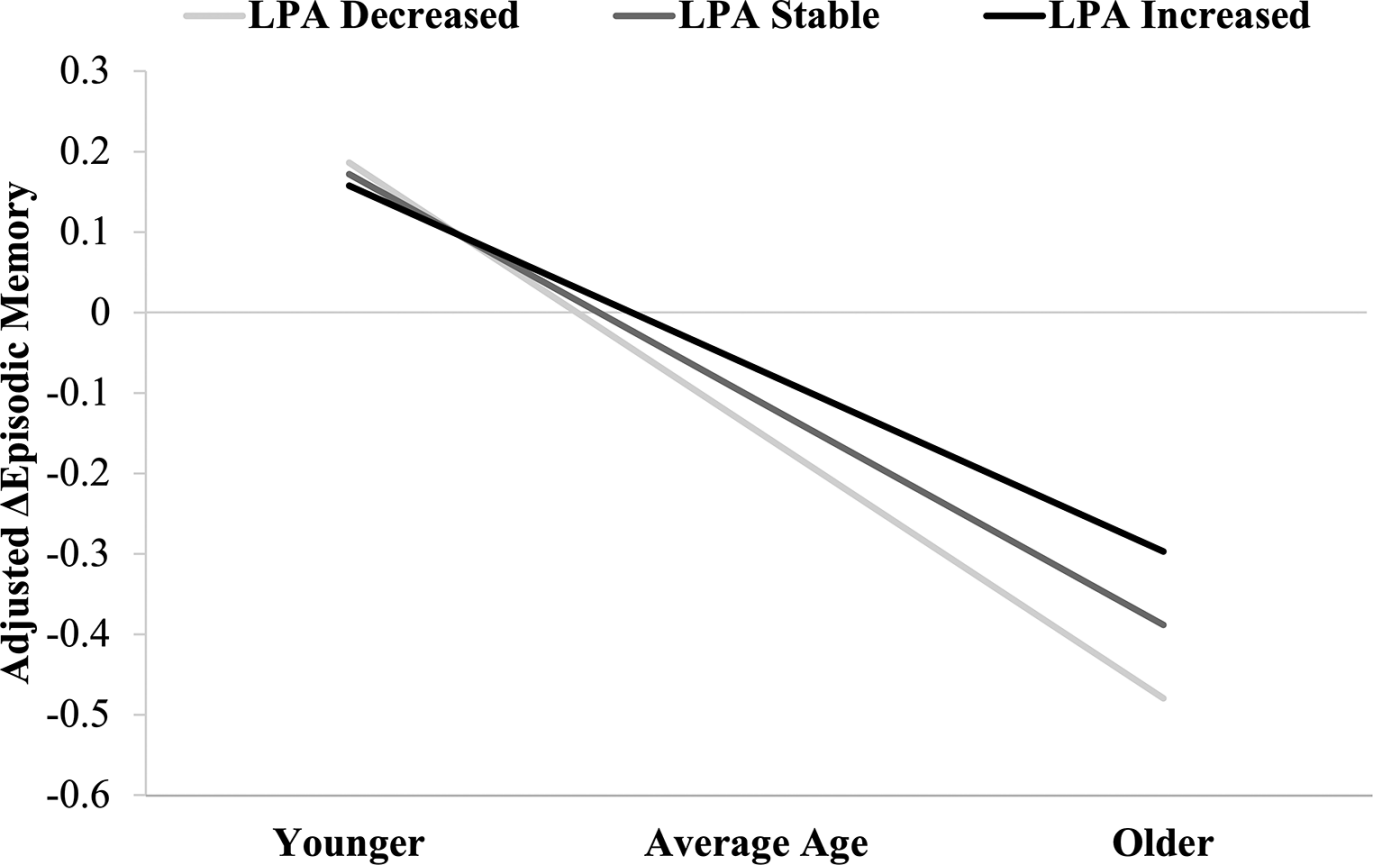
ΔLPA x Age interaction predicting 9-year regressed change in episodic memory. Predicted values were adjusted for average sample declines of − 0.125 units in episodic memory. LPA = light physical activity

Age-moderated associations were observed for both changes in LPA (β = 0.05, *b* = 0.003, *SE* = 0.0011, *p* = .007) and MPA frequency (β = 0.05, *b* = 0.002, *SE* = 0.0009, *p* = .023). Age did not moderate the association between changes in VPA frequency and changes in episodic memory (*p* = .960). Simple slope analyses probed the interactions to assess the influence of LPA and MPA frequency at younger (-1 SD), average (mean), and older ages (+ 1 SD) (Cohen et al., 2013; Hayes, 2017). As expected, results showed the benefits of increasing LPA frequency were pronounced in later life: at younger ages β = − 0.01, *b* = − 0.01, *SE* = 0.021, *p* = .674; at the sample average age β = 0.04, *b* = 0.02, *SE* = 0.014, *p* = .079; and at older ages β = 0.09, *b* = 0.06, *SE* = 0.015, *p* < .001 (see Fig. 3). A relatively similar pattern emerged for MPA, but none of the simple slopes were significant: at younger ages β = − 0.05, *b* = − 0.03, *SE* = 0.017, *p* = .122; β = − 0.01; at the sample average age *b* = − 0.00, *SE* = 0.011, *p* = .794; and at older ages β = 0.04, *b* = 0.02, *SE* = 0.013, *p* = .117.

We further probed the LPA x Age interaction using the Johnson-Neyman spotlight approach for a finer grained analysis (Hayes, 2017). Results showed the influence of LPA was significant at *p* ≤ .05 for adults aged 56 years and older (46% of the analyzed sample). Examining the association across the observed age range of our 33-83-year-old sample revealed that increases in LPA frequency did not predict changes in episodic memory for adults in young adulthood and midlife (aged 33–55). In contrast, the positive and significant association between changes in LPA frequency and corresponding changes in episodic memory became stronger with each passing year for older adults (aged 56–83): from β = 0.04, *b* = 0.03, *SE* = 0.013, *p* = .050 at age 56 to β = 0.17, *b* = 0.11, *SE* = 0.029, *p* < .001 at age 83.

#### Executive functioning

Age only moderated the association between changes in MPA frequency and changes in 9-year executive functioning (β = 0.04, *b* = 0.001, *SE* = 0.0005, *p* = .042). Simple slope analyses showed the benefits of increasing MPA frequency became stronger in later life: at younger ages β = 0.00, *b* = 0.00, *SE* = 0.010, *p* = .972; at the sample average age β = 0.04, *b* = 0.01, *SE* = 0.007, *p* = .069; and at older ages β = 0.08, *b* = 0.02, *SE* = 0.008, *p* = .002. Age did not moderate the association between changes in LPA or VPA frequency and changes in executive functioning (*p*s = 0.288-0.351), although the pattern of simple slopes suggested the benefits of LPA may become slightly stronger at older ages.

## Discussion

MVPA has long been recognized as a means of maintaining physical and cognitive health into old age and remains the focus of public health messaging (Bull et al., 2020). This is unsurprising given that decades of evidence show MVPA is a robust predictor of cognitive aging (Blondell et al., 2014; Guure et al., 2017; Hoffmann et al., 2021). Although these findings have important implications for younger and middle-aged adults, they may be less consequential for older adults who commonly experience age-related physical and functional declines that can limit opportunities to engage in vigorous physical activities (Heckhausen et al., 2019; National Prevention Council, 2016). Our study provides initial evidence that, over and above the influence of more vigorous forms of physical activity, increases in LPA frequency are associated with shallower 9-year declines in episodic memory and executive functioning. Results also advance the literature in showing these more feasible (light) activities, which remain ingrained in everyday life, may become paramount in buffering against episodic memory declines in old age.

Our study is among the first to document that long-term *changes* in LPA predict rates of cognitive decline. Results extend previous research that has focused on the role of LPA levels assessed at a single occasion (e.g., Laurin et al., 2001; Lee et al., 2013; Stubbs et al., 2017; Whitaker et al., 2021) and highlight the importance of capturing the ecological reality that LPA can (and often does) shift over time. Our findings show increases in LPA frequency may have small but meaningful consequences for longitudinal rates of cognitive decline. Contextualized effect sizes suggest that rates of age-related decline in episodic memory and executive functioning were reduced by 50–55% for participants who increased their frequency of LPA by a standard deviation relative to those who remained stable. These findings have implications for the development of evidence-based interventions and point to the potential value of targeting increases in LPA to buffer against cognitive declines. LPA may be an ideal behavioral mechanism to target considering these activities are modifiable, feasible for adults across the lifespan, and remain ingrained in everyday activities for most individuals (Chipperfield, 2008; Chipperfield et al., 2008; Erlenbach et al., 2021).

The present study also contributes to a more nuanced understanding of the developmental circumstances under which LPA may have pronounced consequences. Our findings provide new evidence that increases in these feasible activities may be most protective against memory declines during the stage of the lifespan when they are most needed (in old age). These results extend previous research that provided initial evidence for the benefits of LPA in older samples but did not examine whether this association became stronger in late life. Findings also have encouraging practical implications for older adults considering that our preliminary analyses suggest LPA may be the form of physical activity least susceptible to age-related losses that can undermine MPA and VPA. These findings also have relevance for public health messaging and interventions intended to increase adaptive health behaviors as a means of preserving cognition into old age. Results suggest a greater emphasis on maintaining or increasing more feasible LPAs, such as light walking and light housework, may be warranted in older populations.

Although our study is supported by the use of 9-year data on physical activity and cognitive functioning in a large national sample, it is not without limitations. First, as is typical in large-scale, longitudinal field studies (Yemiscigil & Vlaev, 2021), MIDUS measures of physical activity were based on self-reports. Individuals tend to overestimate their levels of physical activity which can result in some bias when estimating absolute levels (Lee et al., 2011). However, because our primary measures of physical activity were based on *changes* over time, this issue was partially mitigated to the extent that participants who overestimate their activity at one wave may be more likely to do so again at subsequent waves. Future research is nevertheless needed using behaviorally assessed physical activity. Second, changes in our predictor and outcome variables occurred during a largely overlapping time interval because they were assessed at the same waves (Waves 2 and 3). However, the present study does feature a modest time lag given that our predictor variables (LPA, MPA, VPA) were measured approximately one-month prior to our outcome variables at both waves (episodic memory, executive functioning). Nevertheless, the correlational nature of our study precludes making causal inferences about the relationship between LPA frequency and cognitive functioning, which we expect to be reciprocal or bidirectional (Erickson et al., 2022; Robinson & Lachman, 2016). Although we controlled for an array of sociodemographic and health-related factors implicated in cognitive aging, we cannot rule out the possibility that unmeasured or uncontrolled “third variables” could account for the observed relationships. Randomized controlled trials designed to increase LPA frequency are needed to establish that changes in LPA precipitate subsequent changes in cognition (Shadish et al., 2002). Third, the MIDUS sample was largely white and upper-middle class. Further research needed to replicate these findings in racially and socioeconomically diverse samples.

In sum, the present findings provide evidence that increases in the frequency of LPA over extended time periods can have small, but meaningful, implications for healthy cognitive aging. Participants in our national sample who increased their frequency of LPA in midlife and old age experienced less decline in their episodic memory and executive functioning over a 9-year follow-up. Findings also suggest that the benefits of increasing LPA for buffering against losses in episodic memory may become pronounced as individuals age and encounter more barriers to MVPA. Healthcare providers should recommend LPA to their older patients as a feasible mode for engaging in physical activity that may also preserve cognitive function.

## Data Availability

MIDUS data are available from the Inter-university Consortium for Political and Social Research: https://www.icpsr.umich.edu/icpsrweb/ICPSR/series/203.

## Footnotes

1 Raw change scores indicated that participants in our sample exhibited variability in the extent to which they declined, remained stable, or increased in their LPA (25% declined, 52% stable, 23% increased), MPA (36% declined, 28% stable, 36% increased), and VPA (36% declined, 29% stable, 35% increased).
